# A Co-Design-Based Reliable Low-Latency and Energy-Efficient Transmission Protocol for UWSNs

**DOI:** 10.3390/s20216370

**Published:** 2020-11-08

**Authors:** Xiaohui Wei, Hao Guo, Xingwang Wang, Xiaonan Wang, Chu Wang, Mohsen Guizani, Xiaojiang Du

**Affiliations:** 1 College of Computer Science and Technology, Jilin University, Changchun 130000, China; weixh@jlu.edu.cn (X.W.); guohao17@mails.jlu.edu.cn (H.G.); xnwang19@mails.jlu.edu.cn (X.W.); wangchu@jlu.edu.cn (C.W.); 2 Department of Computer Science and Engineering, Qatar University, Doha 2713, Qatar; mguizani@gmail.com; 3 Department of Computer and Information Sciences, Temple University, Philadelphia, PA 19122, USA; dux@temple.edu

**Keywords:** QoS of UWSNs, time-critical aquatic applications, co-design, MAC and routing, transmission protocol, reliable, low-latency, energy-efficient

## Abstract

Recently, underwater wireless sensor networks (UWSNs) have been considered as a powerful technique for many applications. However, acoustic communications in UWSNs bring in huge QoS issues for time-critical applications. Additionally, excessive control packets and multiple copies during the data transmission process exacerbate this challenge. Faced with these problems, we propose a reliable low-latency and energy-efficient transmission protocol for dense 3D underwater wireless sensor networks to improve the QoS of UWSNs. The proposed protocol exploits fewer control packets and reduces data-packet copies effectively through the co-design of routing and media access control (MAC) protocols. The co-design method is divided into two steps. First, the number of handshakes in the MAC process will be greatly reduced via our forwarding-set routing strategy under the guarantee of reliability. Second, with the help of information from the MAC process, network-update messages can be used to replace control packages through mobility prediction when choosing a route. Simulation results show that the proposed protocol has a considerably higher reliability, and lower latency and energy consumption in comparison with existing transmission protocols for a dense underwater wireless sensor network.

## 1. Introduction

The ocean is an important factor for human survival, reproduction and sustainable social development. The total sea area accounts for more than 70% of the Earth. Recently, the field of ocean development has gained tremendous attention from a research perspective. Underwater wireless sensor networks (UWSNs) have been considered as a powerful technique for many time-critical aquatic applications, such as security surveillance, monitoring pollution, submarine tracking and so on [1]. To meet the QoS of these applications, delay, reliability and energy constraints should be satisfied. In other words, it is critical to design a data transmission protocol which can reduce the end-to-end transmission delay and energy consumption, and improve the reliability of transmission for time-critical aquatic applications. The discrepancies between underwater and terrestrial environments further complicate the design of data transmission protocols.

The unavailability of long-distance optical communication leads to most underwater protocols relying on acoustic communication for data transmission. Although underwater acoustic communication has the properties of long acoustic propagation delay and high communication cost, it is possible to satisfy any two of the three above constraints by using separate designed routing or media access control (MAC) protocols. However, the separate design method cannot successfully deal with all three constraints simultaneously in UWSNs.

In order to achieve reliability of transmission, MAC protocols allow the sender and receiver to handshake by sending small control packets (e.g., request-to-send (RTS)/clear-to-send (CTS) [2])) such as channel contention-based carrier sense multiple access/collision avoidance (CSMA/CA). Another common method is to collect information like time division multiple access (TDMA) [3] through a central node to manage the channel. The common feature of these methods is that each pair of nodes to be communicated will require at least one handshake. As for routing protocols, they improve the reliability by selecting multiple relay nodes, creating multiple copies of data or retransmission mechanisms. Sender-based routing protocols (e.g., PULRP [4]) let the sender choose a certain group of forwarding candidates from neighboring nodes as the next-hop. Before sending data packets, the sender broadcasts a control packet to its neighbors for choosing the next-hop. These methods all increase the amount of communication in the network in different ways. However, the transmission cost is very expensive in UWSNs compared with that of traditional MAC protocols in radio communication [5] and this cannot be ignored. In other words, it is difficult to reduce end-to-end delay and energy consumption to maintain reliability of transmission.

In UWSNs, two main methods can reduce the end-to-end latency: reducing the numbers of any manner of communication and reducing the waiting time of nodes. For the first method, ALOHA-based UWAN MAC protocols [6] allow a node to transmit a data frame anytime at its will without any control packets. Receiver-based underwater routing protocols (e.g., depth-based routing (DBR) [7]) do not send any control packets for reservation before data transmission. Senders put their own information in the packet head and directly broadcast the data packet. Receivers utilize specific information (e.g., depth information) in the packet head to decide whether and when to relay this packet or not. Obviously, these two methods cannot guarantee delivery reliability. For the second method, nodes in a centralized-based MAC need to wait for instructions from the central node, and nodes in receiver-based routing need to wait for information from other receivers. In order to reduce the waiting time, this will increase the probability of packet conflicts and thus reduce reliability.

The sources of underwater energy consumption are mainly the number and duration of transmissions. That is, the fewer the transmissions, the lower the energy consumption. At present, ALOHA-based UWAN MAC protocols are undoubtedly the most ideal. Receiver-based protocols reduce energy consumption to a certain extent without additional control packets. However, numerous packets traveling through the network will result in higher energy consumption. The holding time mechanism in DBR and other receiver-based routing protocols can alleviate this problem. Nevertheless, there exists a special case: two non-adjacent nodes receiving packets from a same sender successfully may render the holding time mechanism invalid. Considering this situation, the solution of the multiple copy problem is confronted with new challenges. As discussed earlier, these two kinds of protocol cannot meet the constraint of reliability.

Combining the above points, we found that the reliability of transmission requires time and energy to ensure There is also a certain degree of contradiction between time and energy. In terrestrial wireless sensor networks (TWSNs), the layered structures like MAC and routing facilitate the development, design and troubleshooting of each layer, and can also prevent changes made to one layer affecting other layers. This design concept is based on the speed of optical communication, and the overhead of small control packets in each layer can be ignored. But in UWSN, transmission resources are very scarce and it is advantageous to have control packets on every layer. If we use only one control packet to complete the function of two layers together, this would improve the QoS of UWSN effectively. In addition, the sharing of information between different layers may also reduce the number of communications. Thus, it is worth developing the co-design method underwater.

Co-design in this paper means that routing and MAC work together and share their information with each other. That is, they implement the functionality of MAC and routing jointly. The information from the MAC process provides sufficient information and can be obtained without communicating with neighbors. Latency and energy consumption can be further reduced while maintaining reliability. With the help of routing information, certain strategies can be used during the routing phase to improve the probability of handshake success in MAC. In the routing, the sender can choose the next-hops that have fewer competitors. The acquisition of neighbor information requires additional topology update packets. However, unlike control packets, these topology update packets can be transmitted when the channel is idle so as to affect the transmission as little as possible. Moreover, having neighbor information in the routing phase can effectively alleviate multi-copy issues, thereby reducing energy consumption. In such a case, the co-design method will reduce latency and energy consumption without loss of reliability.

This paper proposes a reliable low-latency and energy-efficient co-design transmission protocol (CDTP) for UWSNs. With the help of the co-design mechanism, the protocol exploits fewer control packets to reduce end-to-end latency and improve the reliability of transmission effectively. At the same time, it alleviates the multi-copy problem to save energy successfully. In the network initialization phase, all nodes will obtain their neighbor information. After the initialization phase, nodes utilize the information in the MAC process (such as the *doppler scale shift* [8]) to estimate the relative speed between nodes and further estimate when their neighbors are out of range. In such cases, control packets previously used in routing can be replaced by a small number of neighbor information update packets through prediction. Under the condition that the neighbor table is available, a sender selects several nodes from its neighbors as the next-hop. In this process it guarantees that selected receivers are adjacent to each other. Before forwarding the data packet, all selected receivers exploit the holding time mechanism in the MAC process to choose one node to forward the packet. By using routing information, CDTP only needs to ensure that at least one node from the chosen neighbors can be received successfully.

In summary, this paper makes two main contributions.

1. *Achieve lower end-to-end latency and energy consumption with fewer control packets while alleviating the multi-copy problem:* CDTP requires less control packet exchange between senders and receivers during routing and MAC, but can also alleviate the multi-copy problem. Using the neighbor information stored in each node, senders select the relay candidate subset of neighbors and coordinate the forwards hop-by-hop. The special case we mentioned before may render the holding time mechanism invalid. Thus, CDTP provides a novel holding time mechanism to alleviate the multi-copy problem and reduce delay. CDTP combines the advantages of both: low latency of the receiver-based protocol and fewer flying packets in the sender-based protocol.
2. *Improve the reliability and efficiency of acoustic communication by using a co-design mechanism:* These two features are often contradictory in the underwater environment. Independent working routing and MAC protocols cannot achieve these features. In routing protocols, however, the conflicts from the MAC process are not considered. This problem is handed over to the MAC protocol. In fact, routing information can help improve the probability of handshake success in MAC. Analogously, it utilizes the information in the MAC process to estimate the relative speed between nodes and to predict when the node is out of range. With the help of updated neighbor information, the communication between nodes will be more reliable.

The remainder of this paper is organized as follows. Section 2 presents design challenges. Section 3 describes the model and problem of the proposed protocol. The specific design of the protocol is discussed in Section 4. Simulation results are provided in Section 5. Related work is reviewed in Section 6, followed by conclusions in Section 7.

## 2. Related Works

In this section, we introduce underwater transmission protocols in turn, which can be divided into three main categories: reliable transmission protocols, low-latency transmission protocols and energy-efficient transmission protocols. Among these, each type of protocol is composed of MAC-layer and routing-layer protocols. A few cross-layer design protocols are also embedded in these three types of protocol.

### 2.1. Reliable Transmission Protocols

JM Jornet et al. [9] proposed the FBR protocol for UWSNs. Before a sender has a data packet to be sent to the destination, it will multicast a request-to-send (RTS) packet with initial power *P* and location information. After calculation, only the nodes that lie within a cone of angle ±θ/2 are considered best hop candidates and will reply to the sender with a clear-to-send (CTS) packet. RACAA [10] creates some reliable end-to-end connected routes from source to destination based on neighborhood information. A node chooses the route with the highest probability of success. However, the fluctuation of the channel is ignored. RECRP [8] further considers the mobility of nodes and exploits cross-layer information to update routing tables. Forwarding nodes are selected based on the routing tables. During the periodic routing update phase, excessive energy will be spent.

Generally in CSMA/CA-based MAC protocols, RTS/CTS handshake protocols are used to handle the hidden terminal problem. This method can improve the reliability of the transmission, and the size of control packets is also very small. However, the underwater transmission time and limited bandwidth mean this method cannot be ignored. In order to reduce the number of RTS/CTS packets, [11] considered the CTS corresponding to the RTS from the source node as both the positive response to the RTS, and the RTS of the CTS sender to one of its neighbors toward the destination of the packet. Each node in an FDMA-based protocol UW-OFDMAC [12] is assigned a dedicated sub-channel according to the receiver location and motion effects to avoid multi-node interference. A sub-channel is further divided into multiple orthogonal subcarriers. The notification frame is needed when receiver location and motion effects are present, and OFDMA is not suitable for underwater because of the scarcity of available channels.

### 2.2. Low-Latency Transmission Protocols

In depth based routing (DBR) algorithms such as [7], EEDBR [13], the sender in these protocols will sense its current depth position relative to the surface and put this value in the packet header. When a receiver receives a packet, it will compare its current depth with the depth value in the packet to determine whether to forward the packet further or not without control packets. However, it is very likely that multiple nodes will forward the same packet and also receive the same packet multiple times. The holding time mechanism is often used in such protocols to alleviate this issue. During this period, the mechanism will discard the packet if it detects the same packet again, otherwise it will forward the packet. If the holding time mechanism cannot avoid the multiple copies, this will result in void communication regions. DVOR [14] is an opportunistic routing protocol that uses the query mechanism to set up distance vectors for all nodes, the sensor nodes having their own distance vector that is determined by the hop-count from the sink. The relay priority for each node can be calculated from the hop-count in the distance vector. The receiver will decide whether to discard or rebroadcast it, and when to rebroadcast it is determined according to its distance vector. DVOR avoids the problems of void regions and long tours, however, simultaneous forwarding by multiple nodes with the same hop-count may cause multi-copy problems. DQELR [15] implements a deep Q network (DQN) algorithm to route adaptively according to network conditions. To improve the performance of transmission, the authors combine unicast and broadcast communications using a hybrid approach. This takes time and adequate data to deploy in different networks. DMR [16] divides the network into four parts and deploys a sink in each part. Compared to the one-sink case in which data needs multiple hops to the sink nodes, multiple sinks will reduce latency. However, the long detour issue may occur inside each part and the new sink will die earlier than other nodes.

ALOHA-based UWAN MAC protocols are the simplest MAC protocols, with which a node transmits a data frame anytime at its will without any control packets. Ref. [6] shows that collision probability is around 0.4 for most traffic arrival rates underwater. Obviously in most cases, this type of protocol cannot be used directly underwater. The protocol [17] consists of a TDMA scheduling scheme for interference reduction and a hierarchical routing scheme for reliable data transmission in UWSNs. The article describes a tightly coupled MAC and routing protocol, which may not work well with other protocols. The optimization of energy costs is not considered. ST-MAC [18] formulates a TDMA-based scheduling problem as an NP-complete vertex coloring problem for a conflict graph. The graph consists of links and their relationships. Through a link-scheduling algorithm it allocates transmission time to nodes. Similarly in [19], which proposes an interference-free graph according to the links between nodes, the two protocols take advantage of the spatial–temporal reuse strategy for the TDMA. Both of these will improve transmission efficiency.

### 2.3. Energy-Efficient Transmission Protocols

The routing protocol in [20] uses the history of successful transmission to the neighbor nodes, the link quality, the residual energy and the buffer space to select next-hop relay nodes. For this reason, the protocol has good next-hop node selection and energy consumption performance. The work in [21] divides the nodes into different levels based on their power level. The nodes can adapt their transmission power based on the network conditions. The power levels and the residual energy of nodes are used to choose the next-hop relay nodes. PULRP [4] and E-PULRP [22] layer the network according to the distance between the sink and nodes. When a sender broadcasts a control packet with its information, the receiver in the lower layer will respond with an ACK. Once the potential relay node is identified, all other nodes can go back to sleep. In this case, the multi-copy problem can be avoided and thus energy can be saved. EDBF [23] uses a depth-based forwarding mechanism to select next-hop with three parameters: depth of the forwarding node, residual energy and forwarding quality. It reduces communication overhead, but in determining forwarding quality, the end-to-end delivery ratio does not improve significantly.

DCC-MAC [24] divides the channel into a common control channel (CCC) and multiple data channels. Nodes in DCC-MAC can adjust the bandwidth of their control channel adaptively by flexibly selecting proper data channels to extend their control channel. It can significantly reduce the collision probability among control messages, thus further reducing data packet collision and energy consumption. However, control messages taking up the data channel will result in increased data transmission time. MC-UWMAC [25] operates on a single slotted control channel to avoid the missing receiver problem and multiple data channels to improve the network throughput. To make it collision-free, MC-UWMAC proposes a grid-based slot assignment procedure on the common slotted control channel and a quorum-based data channel allocation procedure. The channel allocation process requires only the location information of the nodes themselves and does not require the interaction of information between nodes. Further, MC-UWMAC allows multiple data communications along with handshaking on the common control channel to take place at the same time. These two approaches reduce the exchange of control packets and hence the network energy efficiency is improved. However, this multi-channel mode will increase transmission delay underwater, which is not suitable for transmission-intensive scenarios.

## 3. Design Challenges

The design of data transmission protocols in UWSNs is different from terrestrial wireless sensor networks (TWSNs) and presents many challenges [3,26] due to the unique characteristics such as involuntary mobility of underwater nodes and acoustic communication. These characteristics would lead to high latency, high energy consumption and high error probability [27]. Thus, data transmission protocols previously designed for TWSNs need to be redesigned for using in complicated underwater environments. For the most part, protocols in underwater environments require low-latency, energy-efficient and reliable data transmission.

### 3.1. High Latency and Limited Channel Capacity

Data transmission in UWSNs will confront high-latency and low-bandwidth acoustic communication. The acoustic propagation speed (about 1500 m/s in seawater) is much slower than light speed in RF wireless networks. Limited channel capacity and high dynamics of channel quality would also affect the quality of acoustic propagation. Even the transmission of small control packets will also have a certain impact on the end-to-end latency. A quantity of routing protocols lets sensor nodes broadcast control packets to select the next-hop before forwarding data packets. Similarly, control packages are also used in MAC protocols to reserve channels for avoiding the collisions. No matter which layer of protocols, the transmission of control packets will affect the end-to-end latency. More seriously, collision at receivers is more prone to occur because of too many control packets. In addition, long propagation times and flying packets would lead to high bit error rates (BER) then further reduce the reliability of transmission. Once a collision or BER has occurred, senders will typically utilize routing protocols to enable the retransmission mechanism for solving this problem (senders will send the previous packet to its next-hop again), which would further increase end-to-end delay. For these reasons, the number of control packets must be considered to improve the QoS of UWSNs.

### 3.2. Energy Constraints

The energy consumption of sensor nodes in underwater environments is a non-negligible problem. Further, the energy-consumption problem in UWSNs is different from that in TWSNs. Due to the special features of the underwater environment, the sensors in UWSNs are generally equipped with battery power and their relative inaccessibility renders it difficult to replace this limited power supply when exhausted. Energy issues directly affect the lifetime of the entire sensor network. Under the influence of some underwater routing protocols, redundant copies of data packets are inevitably generated for making the transmission more reliable. To prolong the lifetime of UWSNs, redundant copies of data packets among sensor nodes should be reduced without reducing transmission reliability. Thus, the number of redundant copies of packets must be kept low to conserve energy. In addition, the retransmission mechanism and large numbers of control packets previously mentioned will further increase the energy consumption.

### 3.3. Mobility

Objects residing in an underwater environment commonly exhibit passive mobility from water currents or active mobility exhibited by autonomous platforms [28]. Therefore, the neighborhood of a node will constantly change due to this feature of an underwater environment. That is to say, it is difficult for a node to obtain the geographic information of its neighbors. However, frequent neighborhood discovery is necessary in UWSNs to achieve an efficient selection of next-hop candidate nodes. Critically, it is unrealistic to find a fixed routing path with dynamic network topology in a complex underwater propagation environment. The routing process demands frequent updates and maintenance of the routing table, which will undoubtedly increase the end-to-end latency and energy consumption. Due to the long propagation delays, the route information would soon become stale. It is difficult to maintain fresh route information without enough update control packets.

In summary, underwater data transmission protocols require cutting back the number of control packets and reducing the redundant packets to improve the QoS of time-critical aquatic applications. Meanwhile, the protocol needs to overcome the challenges brought by node movement to ensure reliability.

## 4. Problem Statement

### 4.1. Network Model

**Figure 1:**
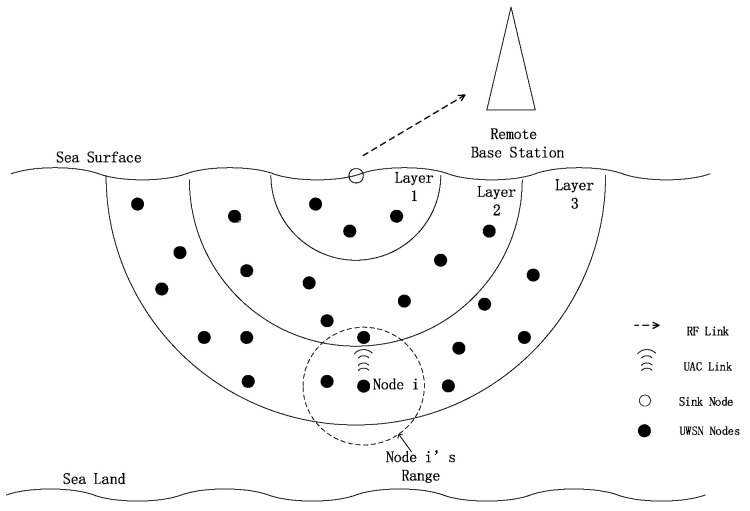
The Network Instruction.

We consider a 3D UWSN architecture consisting of a number of sensor nodes that are randomly and densely deployed in an underwater region, as illustrated in Figure 1. A single sink is located at the center of the network on the water surface. A remote base station is deployed on the ground. Sensor nodes sense and record data, and after that the data are delivered to the sink hop-by-hop. The sink then wirelessly (RF) transmits data to a remote base station. When deploying underwater sensor nodes, a unique ID is given to each node n∈N, where *N* is the set of deployed sensors. During the initialization of the underwater network, sensor nodes are layered as shown in Figure 1. Let $l_{n}$ express the layer number of node *n* (e.g., the layer number of node i in Figure 1 is 3). Each node in the network is assigned to a specific layer number according to its distance relative to the sink. For convenience, the layer number of the sink is assigned to 0.

After network initialization, each node maintains a neighbor table *T* that contains the information of its neighbors. The neighbor table includes neighbor ID, neighbor layer number and two-hop neighbor information. Each node classifies its first-hop neighbors to lower-layer neighbors (LN) and higher-layer neighbors (HN) according to the layer number. The LN of *n* expresses the neighbor whose layer number is lower than node *n*, and HN expresses the opposite.

The following assumptions are made in the design of the scheme to facilitate our research:

1. All sensor nodes are randomly deployed in the network with a random walk mobility model with average speed 1.5 m/s [29].
2. The transmission power and receiver power of the node are fixed. Therefore, the transmission range of each node is also fixed.
3. All sensor nodes have identical initial energy and fixed idling power. When no data packet is sent or received, energy consumption of each node is fixed.
4. The network does not vary at a rate faster than a round trip time and adjacent layers perform data forwarding at different time slices to reduce collision.
5. The network is time-synchronized.

Unlike most depth-based routing scenarios, the sink node can deploy underwater or on the sea surface in CDTP. Sensor nodes can deployed randomly around the sink node to forward the data to the sink node through multi-hop forwarding. CDTP is also suitable for the multi-sink scenario. CDTP uses a one-sink model for convenience of description and is suitable for different scenarios.

### 4.2. The Proposed Protocol

**Figure 2:**
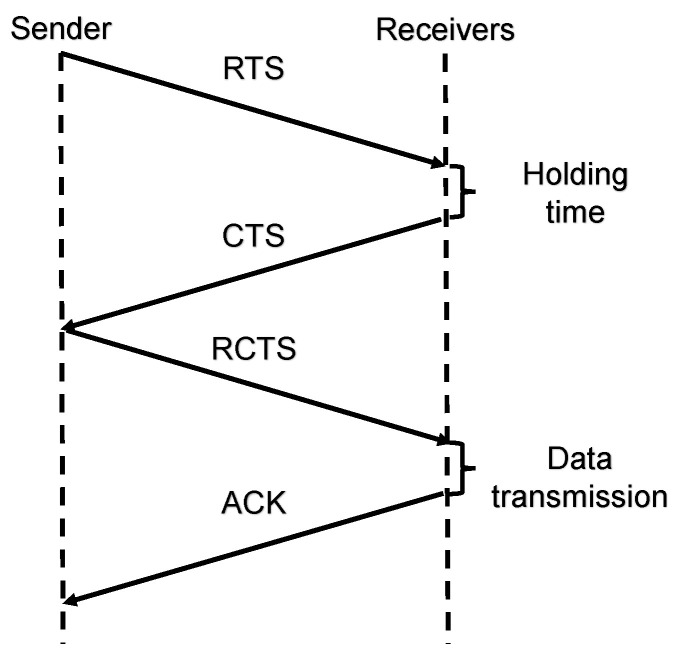
Handshake Process.

In this paper, the distance between any node and the sink is described as the number of layers to the sink (the hop-count from sink). Thus, each node would choose the neighbor that has a lower layer number as its next-hop. CDTP is an approach in which both receiver and sender will participate in the decision making. As shown in Figure 2, a sender first chooses a set of neighbors as its next-hop and puts the selection in a RTS. When selected neighbors receive the RTS, they will return a CTS after a short holding time. Then the sender replies with an RCTS to confirm the receiver and the corresponding data transmission time. These control packets have independent channels and therefore do not conflict with data packets. After screening by the sender and receiver, the best next-hop and transmission time will be selected. It retains the advantages of the two types of routing protocols, which guaranteed the efficiency and reliability of the network.

CDTP consists of two phases: the layering phase and the forwarding phase. In the layering phase, the sink initializes the network by using a layered approach to assign a layer number to each node (e.g., Figure 1). The sink node is considered as a layer 0 node. The sink will initiate the layering process by sending a probe packet. After that, the probe packet will flood to all sensor nodes. Finally, all nodes in the network will obtain their distance from the sink. During this phase, each node records its neighbors’ information in the neighbor table and classifies the neighbors into LN and HN based on this information.

In the forwarding phase, when a node attempts to deliver or forward a data packet to the sink node, it will select a forwarding set from its LN. This node then adds the selection to the RTS and completes the reservation. Only the selected nodes have the priority to reply CTS, other receivers will simply drop the transmission. When a selected node successfully receives the RTS, it will not reply CTS immediately but hold the information for a while to avoid multiple control packets. After this process a RCTS will be the reply from the sender, and the best forwarder will be selected successfully.

In this process, nodes will inevitably receive control packets intended for another node (xRTS, xCTS, xRCTS). In order to prevent conflicts, nodes will have different actions when these are received. $T_{hold}$ denotes the maximum holding time and $T_{round}$ denotes the maximum round-trip time of the control packets. When xRTS is received, the maximum backoff time is set to $T_{hold}+2T_{round}$. If the corresponding RCTS is heard during this time, the backoff is complete. Otherwise the node will wait until the maximum backoff time. The backoff time when xCTS is received is $T_{round}$ until the corresponding RCTS is heard. After xRCTS is received, a successful handshake is completed and the control channel can be occupied again. However, according to the information in the xRCTS, nodes can only start data transmission when the data channel is idle.

During these two phases, there are several challenges that need to be resolved.

1. It is essential to guarantee the neighbor information is available in the case of node movement after network initializing.
2. It is challenging for a node to define the optimal forwarding set from its LN. The optimal subset ought to cut down multi-copies and collision and minimize end-to-end delay simultaneously.
3. It is necessary to determine the calculation of holding time. Scilicet, how long the selected node should hold the CTS to avoid multiple control packets.

We will describe these challenges in detail in next sections.

### 4.3. Multi-Copy Problem

In receiver-based protocols, the routing process will produce multiple copies of a data packet from source nodes to the sink without control packets. This is called the multi-copy problem. Sender-based protocols avoid the multi-copy problem by selecting several specific receivers. There are two major reasons for redundant packets. One is that a node may send the same packet many times. The other is that multiple paths are naturally produced to forward packets. To solve the first problem, the packet history buffer is used to ensure that a node forwards the same packet only once in a certain time interval.

To solve the second problem, the holding time mechanism is widely used in receiver-based protocols, such as DBR [7], DVOR [14]. It is very likely that the multiple neighboring nodes of a node are qualified candidates to forward a packet as the next-hop. If all these qualified nodes have the authority to broadcast the packet, high collision and high energy consumption will occur. Therefore, in order to reduce collisions as well as energy consumption, the number of forwarding nodes needs to be controlled. This prompts a node to queue its receivers by holding the packet for a while to schedule the packet forwarding process. During the holding time, if a qualified node detects the same packet from another qualified node, it will drop the packet to prevent duplicate forwarding. Holding time in DBR and some other depth-based methods is estimated mainly by calculating the value of depth difference between the sender and the receiver. Holding time in DVOR calculates based on the number of hops that the receiver is away from the aggregation node.

**Figure 3:**
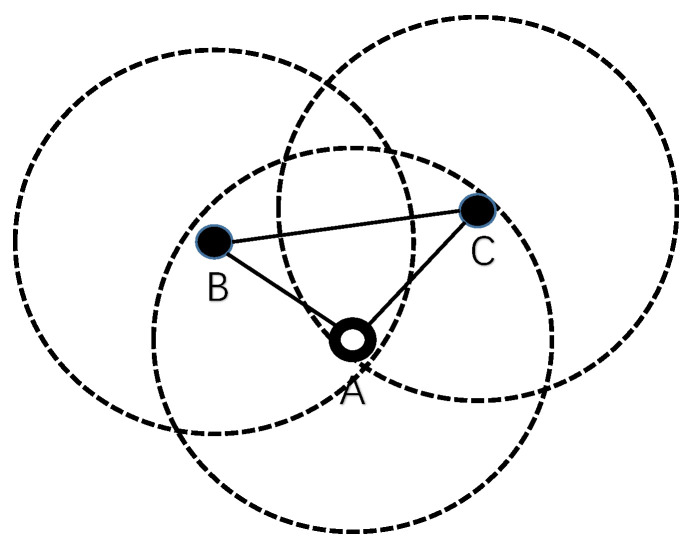
Multi-copy Problem.

Different kinds of holding time mechanism in receiver-based protocols have reduced multi-copy problems effectively. However, the holding time mechanism may be invalid in some cases. For example in Figure 3, there exist nodes *B* and node *C* in the transmission range of node *A*. Obviously, node *B* and node *C* are not within the transmission range of each other. When node *B* and node *C* receive the same packet from node *A*, they will not detect this packet from each other during their own holding time. Node *B* and node *C* will forward the same packet to their neighbors. If this happens once per hop during the transmission to the sink, the final number of received packets will be countless. Eventually, the sink will receive multiple identical packets from multiple paths.

Generally, it is easy to find pairs of nodes like node *B* and node *C* in the transmission range of a node in a dense underwater network. The holding time mechanism may be rendered invalid in such cases. Based on the analysis above, it is necessary to guarantee that qualified nodes will receive packets from each other. In view of this situation, CDTP proposes a different holding time mechanism in the design of the protocol.

### 4.4. Dynamic Routing Problem

After the layering phase, each node will obtain their neighbor table *T*. In the neighbor table, all neighbors are classified as lower-layer neighbors (LNs) and higher-layer neighbors (HNs). As long as each node transmits packets to its LNs, all packets will eventually be transmitted to the sink node. Therefore, when node *n* has a packet to forward, it will choose several neighbors from its LN as the next-hop. Let $F_{n}=\left\{f_{n}^{1},f_{n}^{2},...,f_{n}^{j}\right\}$ express the forwarding set of *n*, where $f_{n}^{j}$ is the $j^{th}$ LN of node *n*. Node *n* is going to select one or more nodes from $F_{n}$ as its next-hop. Let selection strategy $S_{n}=\left(S_{n}^{1},S_{n}^{2},...,S_{n}^{j}\right)$ indicate the choice of node *n* to all LN in $F_{n}$, where binary $S_{n}^{j}\in \left\{0,1\right\}$ denotes the choice of node *n*. $S_{n}^{j}=1$ means node *n* will choose node *j* as its next-hop, and 0 otherwise.

According to the forwarding set $F_{n}$ and the neighbor table *T*, a j∗j adjacency matrix *E* is constructed, where *E* is created to express the relationship connectivity between nodes in $F_{n}$. Let $f_{n}^{\alpha }$ be one of the vertices $v_{\alpha }$ in adjacency matrix *E*, and binary $e_{\alpha \beta }\in \left\{0,1\right\}$ express the neighbor relationship between vertex $v_{\alpha }$ and vertex $v_{\beta }$. The element $e_{\alpha \beta }$ is 1 when vertex $v_{\alpha }$ and vertex $v_{\beta }$ are neighbors, and zero when they can not detect each other.

Due to the existence of the neighbor table, no control packet is needed before sending a data packet. Strategy $S_{n}$ will select multiple nodes as the next-hop, as long as one node in $S_{n}$ forwards the packet regarded as a successful transmission. Thus, CDTP will not perform collision detection for each pair of nodes and no longer sends control packets for the MAC-layer. These two aspects will effectively reduce the end-to-end latency. After reducing the end-to-end latency, the selection strategy $S_{n}$ ought to maximize one-hop reliability by avoiding collision and minimize the multi-copy problem to maximize the system performance. This can be formulated as the following optimization problem:

(1) $$\begin{array}{cc} \; & \arg\max_{S_{n}}P^{n\to S_{n}} \end{array}$$

This is subject to the following constraints:

(2) $$\begin{array}{cc} \; & \sum_{\beta =2}^{j}\sum_{\alpha =1}^{\beta -1}S_{n}^{\alpha }S_{n}^{\beta }e_{\alpha \beta }/\sum_{\beta =2}^{j}\sum_{\alpha =1}^{\beta -1}S_{n}^{\alpha }S_{n}^{\beta }=1 \end{array}$$

(3) $$\begin{array}{cc} \; & S_{n}^{\alpha },S_{n}^{\beta },e_{\alpha \beta }\in \left\{0,1\right\} \end{array}$$

$P^{n\to S_{n}}$ is the probability that at least one chosen forwarder of node *n* under strategy $S_{n}$ receives a packet successfully. However, it is hard to estimate the value of $P^{n\to S_{n}}$ due to the complex underwater environment and the randomness of the packet generation. To estimate and maximize the value of $P^{n\to S_{n}}$, we convert this problem to a multi-objective optimization problem in the next section. During this process, the neighbor information between nodes is fully utilized to maximize the transmission reliability hop by hop.

Equation (2) ensures that the chosen nodes in $S_{n}$ can detect each other. For any pair of selected nodes α and node β in $S_{n}$, $e_{\alpha \beta }$ all equal to 1 indicates that the nodes in the selection strategy satisfy the above condition. This will avoid the special case resulting in the multi-copy problem mentioned earlier.

## 5. Design of Data Transmission Protocol

### 5.1. Network Initialization

In the layering phase, the sink node starts to initialize the network by propagating a probe packet. The sink node is assigned to be the layer 0 node. The sink will broadcast a probe packet, which contains its layer number and ID. The nodes that receive this probe will assign themselves as the layer 1 nodes. These nodes will increment the layer number, change the ID to their own and then propagate the probe packet after waiting for a certain time. Once these probe packets reach all the nodes in the network, each node will obtain a layer number depending on its hop from the sink.

During this process, each node records the ID of its neighbors and the pairwise distances from them (the distance is calculated based on the path attenuation model that will be mentioned later). After building its own neighbor table, each node puts its neighbor table into the probe packet before propagating it. That is, probe packets contain the layer number, ID and neighbor table of the current node. Each node will maintain the knowledge of two-hop connectivity after receiving a probe packet from its neighbor.

When the above process is completed, the probe packet will be broadcast from the sink once again. One reason is that the first transmission of the probe packet cannot help the node to obtain complete two-hop neighbor information. When a node decides to forward a probe packet, it may not receive a probe from all neighbors. The other reason is that two probe packets can regulate the layer numbers of all nodes. In order to avoid loops, the two probe packets will be sent after an interval, and each node will only forward a packet once in different time periods. After initialization, all nodes obtain enough information which will be used in the forwarding phase. If each node in its particular layer supposes it will forward a data packet to its LN neighbors, the packet will finally be transmitted to the sink. In other words, the distance between any source node and the sink is over an equal number of hops.

Due to the node movement, the layer numbers of all nodes need to be updated frequently. Whenever a node receives a data packet, it will broadcast an ACK in a data channel. This is considered as a probe packet for the re-layering process for its neighbors. A node may receive multiple probe packets from different layers, but it will record the layer number as the lowest layer number plus one. Whenever a sensor node detects three data packets from a lower layer, say *q*, and does not hear from layer q−1 simultaneously, then the node will assign itself as layer q+1. For the purpose of avoiding glitches [17], the nodes reassign their layer numbers every three times when they detect the probe from the lower layer. It is assumed that binary phase shift keying (BPSK) [20] is used to modulate the underwater acoustic data to avoid high bit error rates (BER).

### 5.2. Dynamic Routing Strategy

In this section, we will convert the dynamic routing problem to a multi-objective optimization problem. The value of $P^{n\to S_{n}}$ will be estimated depending on the neighbor information stored in each node. We extracted three main factors affecting the value of $P^{n\to S_{n}}$ from the neighbor information, which are the number of competitors, the number of chosen forwards and the attributes of competitors.

**Figure 4:**
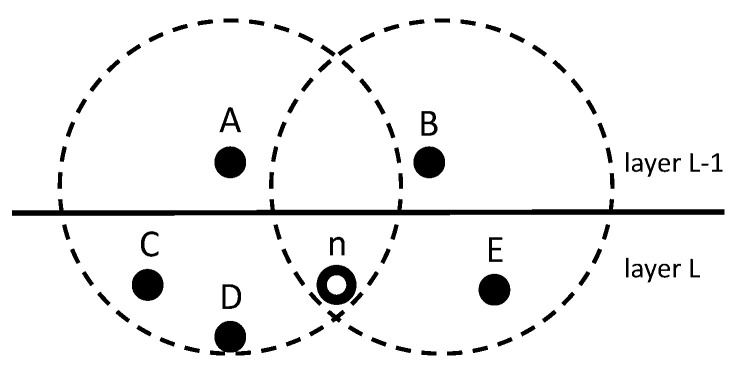
description of the competitor set.

**Figure 5:**
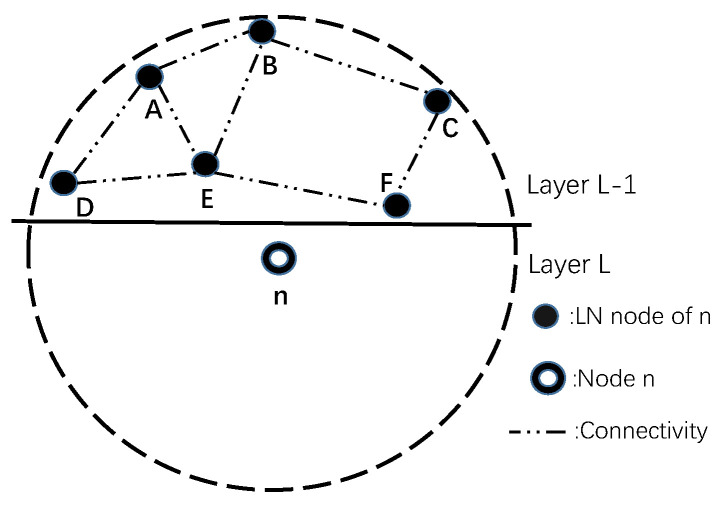
Node n and its forwarding set.

**Figure 6:**
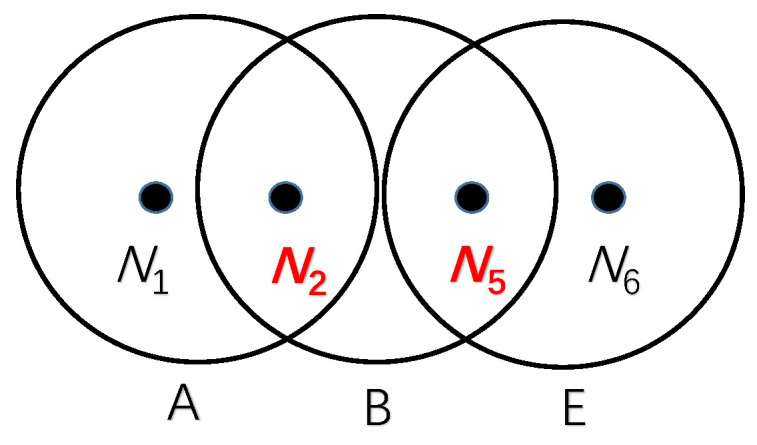
Relationships between the competitors.

1. *Number of Competitors*: According to the forwarding set $F_{n}=\left\{f_{n}^{1},f_{n}^{2},...,f_{n}^{j}\right\}$, node *n* has *j* qualified candidates that can complete the forwarding mission. For each node in $F_{n}$, it may also exist in another node’s forwarding set. In other words, a candidate belonging to node *n* may have multiple HNs besides *n*. Each node in $F_{n}$ may have several HNs, which will affect the transmission of node *n*. All the HNs brought by the nodes in $F_{n}$ will be considered as the competitors of *n*. If these competitors and *n* send packets at the same time, collision may occur at some specific receivers and eventually result in a transmission failure.
  After this analysis, our defined competitor set of node *n* is $C_{n}=\left\{C_{n}^{1},C_{n}^{2},...,C_{n}^{j}\right\}$. The elements in the set correspond to the nodes in $F_{n}$, with each element representing a competitor subset. Competitor subset $C_{n}^{j}=\left\{C_{n}^{j1},C_{n}^{j2},...,C_{n}^{jk}\right\}$ expresses the competitors brought by the $j^{th}$ LN of *n*, where $C_{n}^{jk}$ is the $k^{th}$ HN of the $j^{th}$ LN of node *n*. Figure 4 describes an example of the competitor set belonging to node *n*. Node *A* and node *B* are in the $F_{n}$ of *n*. Obviously, except for node *n*, nodes *C* and *D* may send packets to *A*, and node *E* may send packets to *B*. From the perspective of node *n*, nodes C,D and *E* are regarded as competitors brought by nodes *A* and *B*, respectively. There is no doubt that node *n* is prone to choosing node *B* as its next-hop, which will bring fewer competitors. Fewer competitors means less conflict, which will also improve the value of $P^{n\to S_{n}}$.
2. *Number of Chosen Forwarders*: in addition to the number of competitors, the number of chosen forwarders will also affect the value of $P^{n\to S_{n}}$. In different selection strategies $S_{n}$, there will be different sizes of chosen forwarders. Imagine an extreme situation, where $S_{n}$ selected all nodes in $F_{n}$ as its next-hop, which would maximize the value of $P^{n\to S_{n}}$. That is to say, in the case of satisfying Equation (2), the sender is prone to choosing as many receivers as possible. For example, we choose {A,B,C} as a selection strategy, which satisfies Equation (2). This means that {A,B}, {B,C}, {A,C} will also satisfy the selection strategy. No matter how many competitors the extra node brings, one more candidate will always improve the value of $P^{n\to S_{n}}$. An extra competitor will not affect the original node. In summary, the number of chosen forwarders is proportional to the value of $P^{n\to S_{n}}$.
3. *Attributes of Competitors*: the numbers of competitors and chosen forwarders are not enough to determine the probability of conflict. It is difficult to judge when the above two parameters of the two different selection strategies are equal. Thus, it is necessary to explore the impact of different competitors on conflict.
  Suppose there is a node *x* at a very small distance from *n*, and they even have the same neighbor table. However *n* changes the selection strategy $S_{n}$, node *x* will always affect the transmission of node *n*. It can be argued that each node in $F_{n}$ will also receive a data packet from node *x* constantly. In this case, we believe that node *x* is a strong competitor to node *n*. We will quantify the competitiveness of the set of competitors for each node according to the degree of overlap of their neighbors.
  An example for node *n* and its forwarding set $F_{n}=\left\{A,B,C,D,E,F\right\}$ is shown in Figure 5. Nodes A−F are distributed in the layer L−1, and node *n* is distributed in the layer *L* and will choose several nodes from A−F as its next-hop. The dotted lines between A−F indicate whether they are within communication range of each other, which expresses the neighbor relationships of $F_{n}$. The competitor set brought by forwarders A−F is shown in Table 1. Nodes $N_{1}-N_{8}$ are deployed in the same layer as node *n* (in order to prevent confusion, this is not marked in Figure 5).
  In order to facilitate the explanation of the attributes of competitors on node *n*, we first choose {A,B,E} as the selection strategy that satisfies Equation (2). According to Table 1, if nodes $N_{1}$ and *n* are sending a data packet at the same time, a conflict will occur at node *A*, but will not affect nodes *B* and *E*. Let Figure 6 denote the relationship between the competitors $\left\{N_{1},N_{2},N_{5},N_{6}\right\}$ and chosen forwarders {A,B,E}. Nodes $\left\{N_{1},N_{2}\right\}$, $\left\{N_{2},N_{5}\right\}$ and $\left\{N_{5},N_{6}\right\}$ are brought by *A*, *B* and *E* separately. Nodes $N_{2}$ and $N_{5}$ are at different intersections of two nodes, which coexist in the competitor sets of two nodes. Briefly speaking, if $N_{2}$ and *n* send a data packet simultaneously, *A* and *B* will not receive the packet successfully because of a conflict. However, *E* can detect node *n* in this circumstance. Considering a worst-case scenario, if nodes $N_{2}$, $N_{5}$ and *n* send a package simultaneously, none of the nodes in {A,B,E} can receive the packet, which will cause the selection strategy to fail. After calculation, at least two competitors can cover all chosen forwarders. In other words, less than two arbitrary competitors and *n* can send packets simultaneously this will not cause strategy {A,B,E} to become invalid.
  After the analysis of the example above, we define a *Conflict Competitors Set:* under the selection strategy $S_{n}$, the competitors who will cause a forwarding failure (all nodes in $S_{n}$ influenced by competitors) when they send packets simultaneously with *n*, which is a part of the permutation and combination of $\left\{N_{1},N_{2},N_{5},N_{8}\right\}$ in the above example. The set with the smallest number of elements among all conflict competitor sets will be considered as the minimum conflict competitors set. In the above example, the minimum conflict competitors set is $\left\{N_{2},N_{5}\right\}$. Let $MC_{S_{n}}$ denote the the minimum conflict competitors set calculated from $S_{n}$. The better selection strategy is supposed to have a larger size of $MC_{S_{n}}$. The reason is that a larger size of $MC_{S_{n}}$ can tolerate more nodes sending data packets at the same time. According to the above definition, we can also calculate that the size of the minimum conflict competitors set in selection strategy {A,D,E} is 3($\left\{N_{1},N_{3},N_{5}\right\}$). Obviously, three nodes without the intersection of competitors will perform better. Above all, the attributes of competitors can be quantified by the size of the minimum conflict competitors set.

Considering the impact of the above three aspects on the conflict, the optimization problem can be expressed as:

(4) $$\begin{array}{cc} \; & \arg\max_{S_{n}}|MC_{S_{n}}| \end{array}$$

(5) $$\begin{array}{cc} \; & \arg\max_{S_{n}}-|S_{n}^{1}C_{n}^{1}\cup S_{n}^{2}C_{n}^{2}\cup ...\cup S_{n}^{j}C_{n}^{j}| \end{array}$$

(6) $$\begin{array}{cc} \; & \arg\max_{S_{n}}\sum_{\alpha =1}^{j}S_{n}^{\alpha } \end{array}$$

which are subject to the following constraints:

(7) $$\begin{array}{cc} \; & \sum_{\beta =2}^{j}\sum_{\alpha =1}^{\beta -1}S_{n}^{\alpha }S_{n}^{\beta }e_{\alpha \beta }/\sum_{\beta =2}^{j}\sum_{\alpha =1}^{\beta -1}S_{n}^{\alpha }S_{n}^{\beta }=1 \end{array}$$

(8) $$\begin{array}{cc} \; & S_{n}^{\alpha },S_{n}^{\beta },e_{\alpha \beta }\in \left\{0,1\right\} \end{array}$$

Equation (4) finds the maximum value of the minimum conflict competitors set under different $S_{n}$, where $MC_{S_{n}}$ is the minimum conflict competitors set in $S_{n}$. Equation (5) finds the minimum competitor set from all potential competitors of node *n* under the strategy $S_{n}$, where $C_{n}^{j}$ means the competitors brought by node *j*. In order to unify the format, we converted the symbol for Equation (5). Equation (6) is the maximum for the total numbers of selected nodes in $S_{n}$. This means that when more forward nodes are selected, the transmission success rate is higher. To calculate the optimal strategy $S_{n}$, we need to deal with all three optimization goals at the same time. The number of competitors will increase with the number of forwarders, and the number of competitors and forwarders will affect the size of the minimum conflict competitors set. Therefore, an algorithm needs to be designed to solve these three optimization goals together.

Algorithm 1 can be used to solve the optimization problem above. The algorithm takes as its input all the information from the neighbor table of node *n*. Based on such information, lines 1–4 create a forwarding set $F_{n}$, an adjacency matrix *E* and a candidate set Can. All combinations of the nodes in $F_{n}$ are stored in the candidate set. The eligibility of these candidates is then judged according to Equation (7) (line 5–9). After traversing the qualified candidates set, lines 10–20 calculate the size of the minimum conflict competitors set as $Counter_{i}$ by finding the competitors with the most coverage candidates in turn until all candidates are covered. After calculating the size of the minimum conflict competitors set, lines 21–24 pick the best candidates according to the three different optimization targets. Most directly affecting the probability of conflict is Equation (4). It contains more complex neighbor relationships than the other two expressions. Therefore, this optimization target needs to be satisfied first. Generally, more candidates bring more competitors, and every additional candidate determined will increase the stability of the transmission. For this reason, Equation (6) has a higher calculation priority than Equation (5). Eventually, the algorithm will calculate the filtered candidate set $Can_{0}$ as the optimal selection strategy $S_{n}$.
**Algorithm 1** Dynamic Routing Strategy**Input:** neighbor table T**Output:** optimal selection strategy $S_{n}$ 1: **Find** forwarding set $F_{n}$ from *T*, size of $F_{n}$ is *j* 2: **Create**
j*j adjacency matrix *E* 3: **Create** Vector Counter 4: **Find** all combinations of the elements in $F_{n}$, push into Candidate set Can, size of Can is ε 5: **for**
i=0→ε
**do**
 6:     **if**
$Can_{i}$ does not satisfy Equation (7) **then**
 7:         **Delete**
$Can_{i}$ from Can
 8:     **end if**
 9: **end for**
10: **for**
i=0→ε
**do**
11:     **Find** all competitors of $Can_{i}$, push into competitor set $Com_{i}$
12:     $item1\leftarrow Can_{i}$
13:     $item2\leftarrow Com_{i}$
14:     $Counter_{i}\leftarrow 0$
15:     **while**
sizeof(item1)=0
**do**
16:         **Find** Max element in item2 and **Delete** it 17:         **Delete** element in item1 which has the found Max element 18:         $Counter_{i}++$19:     **end while**
20: **end for**
21: **Find** Max element in Counter and **Delete** other corresponding elements of Can22: **Find** Max $sizeof(Can_{i})$ and **Delete** other elements 23: **Find** Min $sizeof(Com_{i})$ and **Delete** other corresponding elements of Can24: $S_{n}\leftarrow Can_{0}$

After selecting an optimal selection strategy, node *n* will insert the strategy into the packet head and then broadcast it out. Only the nodes in the strategy have the permission to forward the packet, others will simply drop the packet when the packet is received. After holding the packet for a while, the nodes in the strategy spontaneously choose the best node to which to forward the packet. In the next section, we will discuss how to calculate the holding time and the spontaneous selection strategy of nodes.

### 5.3. Holding Time Calculation

The dynamic routing strategy in CDTP provides a selection strategy that contains several neighbors of a node. In our network, the number of hops from the source node to the sink is fixed, and each node in the selection strategy can reach a lower layer. Therefore, for a sender, no matter who the next-hop is, the number of hops to the sink can be guaranteed to be the same. In order to reduce the holding time of nodes and further reduce the end-to-end delay in CDTP, forwarders prefer to elect the node which can forward a packet to its next-hop earliest. In other words, the receiver with the minimal distance from the sender should forward a packet first. The selected receiver under this strategy tries to prevent other neighboring nodes from forwarding the same packet to reduce energy consumption. That is, among the chosen nodes (under selection strategy $S_{n}$), the one that is closer to node *n* has a higher forwarding priority. Selected forwarders and node *n* know the distance information between each other. Then node *n* sorts all forwarders based on their distance to itself.

In Figure 5, {A,B,E} is the optimal selection strategy depending on algorithm 1. According to their distance from node *n*, the forwarding priority is E>A>B. The holding time that is calculated by these nodes will increase with increasing distance from node *n*. Node *E* will reply CTS immediately after receiving an RTS. Therefore, the holding time of node *E* is 0. When nodes *A* and *B* receive the RTS, node *E* should have already received it. If node *E* has successfully received an RTS and forwarded CTS, nodes *A* and *B* should wait until detecting CTS from *E* to avoid multiple flying CTS packets. The holding times of *A* and *B* depend on the distance between *E* and themselves. If *A* and *B* cannot detect the CTS from *E*, node *E* will be considered as a failure, and they will reply CTS on their own. Let $t_{EA}$ and $t_{EB}$ express the transmission time between E,A and E,B. The holding times of *A* and *B* will be $t_{EA}+\xi$ and $t_{EB}+\xi$, respectively, where ξ is a short protection time. The protection time is used to prevent changes in the distance between nodes due to node movement.

In short, the node closest to the sender will reply immediately, and the holding time for other nodes is the transmission time between the closest node and themselves plus a short protection time.

### 5.4. Network Maintenance and Updating

After network initialization, each sensor node maintains a neighbor table which includes the knowledge of its two-hop neighbors and their pairwise distances. To estimate the distance between a sender and its neighbor nodes, we combine the path attenuation model [29], the channel strength and the transmission strength that are obtained from the received data packets. The path attenuation model is

(9) $$A\left(l,f\right)=A_{0}l_{k}a\left(f\right)l$$

where *f* is acoustic communication frequency, *l* is the distance between two nodes, $A_{0}$ is a unit-normalizing constant, *k* is a propagation factor that represents the geometry of propagation, here k=1.5 [30], and a(f) is the absorption coefficient. If expressed in dB, the underwater acoustic path loss is

(10) $$10\log A(l,f)/A_{0}=k10\log l+l10\log a(f)$$

Defined by the Thorps formula [31], the following form is often used for low-frequency signals.

(11) $$10\log a(f)=0.002+0.11f^{2}/\left(1+f^{2}\right)+0.011f^{2}$$

If a hydro-acoustic signal of frequency *f* is transmitted with power *P* along an unblocked path, the received signal strength is P/A(l,f) [8], so we can roughly estimate the distance between two nodes by the received signal strength.

The relationship between Doppler scale shift and relative velocity between any pair of nodes is v=ac, where *v* is the relative velocity, *a* is the Doppler scale shift, and *c* is the sound speed. Relative velocity estimations based on Doppler shifts can achieve a promised performance from the obtained data. For example, [32] reached a 0.1 m/s deviation for node speed with a maximal velocity of 5 m/s. We leveraged the Doppler measurement to estimate the relative velocity.

Now we have the method to calculate the distance and the relative velocity between every two connected nodes. As mentioned before, nodes have constant transmission and receiver power, which means the attainable range is unchanged. Based on this feature, a node can estimate the time when its neighbors will be out of range. If a node has not received a package from a neighbor during the estimated time, the corresponding entry is removed from the neighbor table and the node broadcasts the updated neighbor table to its neighbors. Otherwise, the node will update the relative velocity and the distance about this neighbor using the current information from recently received packets.

There may be a situation where a neighbor is in the neighbor table when the node sends the data packet, however this neighbor actually moves out of the transmission range of the node. In this case, the neighbor table of the node is not fresh anymore and this neighbor can no longer receive packets from the node. In addition, the conflict between nodes may lead to a forwarder’s failure to receive the packet. If all chosen forwarders fail, we call this a transmission failure. We therefore put a failure counter on every node to record the number of transmission failures. If the number of failures counted for the node *n* is more than 3, it will activate a partial update by broadcasting a probe packet to its one-hop neighbors. The probe packet will not cause flooding like network initialization and the neighbors of the node will not forward probe packet again.

In summary, when a neighbor is predicted to be out of range or the failure count of the neighbor is more than 3, the node will perform a partial update. A global update will be triggered periodically.

## 6. Simulation and Performance Evaluation

In this section, with the help of the NS2 simulator and Aquasim [33] in NS2, we evaluate the performance of our proposed protocol by comparing it with DBR, VBF and DVOR, which were proposed in [7,22,34], respectively. In order to compare the overall transmission performance, we have equipped these routing protocols with corresponding MAC protocols. We also simulated the protocol using a Monte Carlo method. All numerical simulation results were obtained after 100 calculations.

### 6.1. Simulation Settings

Sensor nodes are randomly deployed in an underwater spherical 3D region of 300m×300m×300m. The sink node is deployed at the water surface (the coordinate is (0,0,300)). A random walk mobility model with average speed of 1.5 m/s [29] is used in the simulation. Each sensor node randomly selects a direction and moves to the new position with a random speed between the minimal speed and maximum speed, which are 0.5 m/s and 3 m/s, respectively. There is a source node deployed at the bottom of water (the coordinate is (0,0,0)). We assume that the sink node and the source node are stationary once deployed. Data packets are generated by the source node periodically with a packet size of 50 bytes. The average package-generating interval is set at 2 s. We set the simulation time as 300 s and the initial energy of every node as 10,000 J. To better simulate the energy consumption, we set the transmitting and receiving power as 2 and 0.75, respectively. The idling power of every node is set to 0.008. The maximum range of one-hop transmission is 100 m.

We use the following metrics to evaluate the performance of protocols.

1. *Packet Delivery Ratio*: is defined as the sum of the different ID packets received by the sink ($P_{dif}$) divided by the total number of packets generated at the source node ($P_{gen}$), $P_{dif}/P_{gen}$. Although a packet may reach the sink multiple times, these redundant packets are counted only once in $P_{dif}$.
2. *Average Multi-copy Packets*: is defined as all of the packets received by the sink ($P_{all}$) divided by the number of packets generated by the original node ($P_{gen}$), $P_{all}/P_{gen}$. Considering that multiple copies of a packet may reach the sink, the result may be greater than one.
3. *Average End-to-End Delay*: for each packet *p*, the end-to-end delay for packet *p* is the moment a packet arrives at the sink ($T_{arr}^{p}$) minus the moment it is generated ($T_{gen}^{p}$), $T_{arr}^{p}-T_{gen}^{p}$. Therefore, the average end-to-end delay is $\sum \left(Tparr-Tpgen\right)/P_{dif}$.
4. *Total Energy Consumption*: is defined as the initial energy of all nodes in the system ($E_{ini}$) minus the total remaining energy at the end of the experiment ($E_{rem}$), $E_{ini}-E_{rem}$.

### 6.2. Comparison with VBF, DVOR and DBR

Now we compare CDTP with DBR [7], DVOR [14] and VBF [22]. In the set of simulations, we set the MAC protocol to R-MAC [35] for DBR, DVOR and VBF. For CDTP, we do not set any MAC protocol. The MAC protocol is preset in Aquasim [33]. As we discussed in the previous chapters, an ALOHA-based protocol cannot be used well in the previous protocol. During the simulation, we try to deploy the ALOHA protocol in DBR, DVOR and VBF. However, the sink node cannot receive any packets in our simulation settings. CDTP utilizes the co-design method to avoid conflicts successfully. Meanwhile, CDTP can also utilize R-MAC but with little delay and energy.

**Figure 7:**
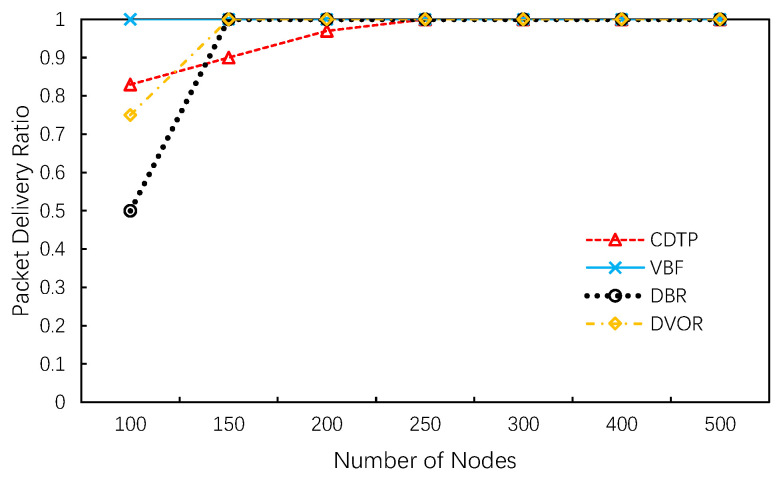
Packet delivery ratio.

From Figure 7, we notice that the packet delivery ratio of CDTP and VBF cannot reach one without enough nodes. This is because there may be isolated nodes in a sparse network that are unable to find the next-hop. With the increase of nodes in the network, this phenomenon will be significantly reduced as shown in the figure. As for DBR and DVOR, the void region in an extremely sparse network makes the delivery ratio lower than CDTP. However, the delivery ratio of these two protocols reaches 1 faster than CDTP and VBF. The closer the delivery ratio gets to 1, the more reliable it proves to be. Packets in CDTP are always forward to lower layers, however in sparse networks some nodes may have neighbors but not lower-layer neighbors.

**Figure 8:**
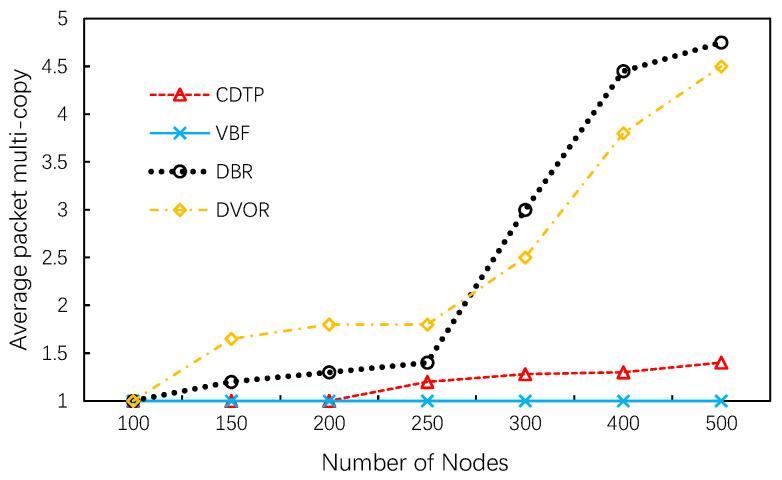
Average multi-copy packets.

According to Figure 8, we observe that the multi-copy packets ratio of CDTP and VBF is close to one. In other words, almost all packets are successfully transmitted to the sink without redundancy. Another point to note is that in DBR and DVOR, the packet delivery ratio increases as the number of nodes increases. The multi-copy packets ratio is more than 4 when 500 sensor nodes exist in the system. Scilicet, for each packet, at least three other identical data packets are transmitted between nodes and eventually transmitted to the sink. This phenomenon illustrates that receivers in DBR and DVOR may be non-adjacent and will not be prevented from forwarding by the holding time mechanism. Simulation results have shown that selection strategies in CDTP can avoid this problem and thus reduce the occurrence of this phenomenon. Fewer multi-copies means less energy consumption.

**Figure 9:**
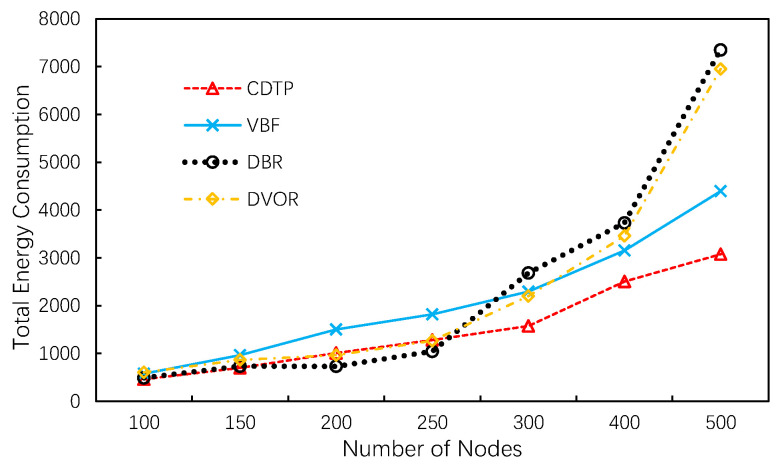
Energy consumption.

The total energy consumption for different numbers of sinks are shown in Figure 9. We observe that CDTP has a higher overall energy efficiency when compared with other protocols. As the figure shows, the total energy consumption of VBF is always higher than CDTP. The reason is that in VBF, once a source node initiates a transmission, it first sets up a coordinate system originated at itself and floods a data ready packet into the network. Conversely, in the case when the numbers of nodes are 200 and 250, the total energy consumption of DBR is slightly lower than CDTP. This is because the percentage of energy consumption in network topology initialization and maintenance operations in CDTP is greater when there are fewer nodes. However, the multi-copy problem decreases as the number of nodes increases, allowing us to save energy. Combining the two points above, CDTP eventually performs well in respect of total energy consumption.

**Figure 10:**
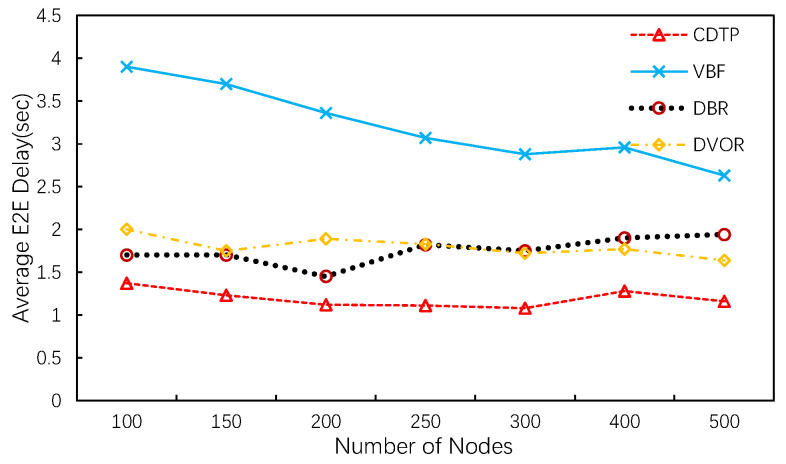
Average E2E Delay.

Figure 10 shows that CDTP achieves the lowest end-to-end delay among the three protocols. Compared to VBF, the end-to-end delay of CDTP shows a significant reduction. This is because VBF tries to find the shortest path from the source node to the sink along the virtual vector between them. As mentioned before, the sink floods a data ready packet before it initiates a transmission. Then, in the process of searching for the shortest path, the node holds the packet for a time interval to wait for a better forwarding option. These strategies increase the transmission delay of the packets. Compared to DBR and DVOR, CDTP transfers the calculation of holding time to the MAC process. The novel holding time mechanism allows each node in CDTP to find the nearest next-layer neighbor to minimize one-hop transmission and waiting time. On the other hand, the average transmission path of CDTP is shorter than for DBR and DVOR because of the hierarchical structure of our network topology.

## 7. Conclusions

In this paper we have proposed a novel co-design-based data transmission protocol to achieve low-latency, energy-efficiency and reliability for UWSNs. Based on the co-design method, CDTP not only maintains the multi-layer structure and neighbor information of the network, but assists in conflict avoidance. With the help of neighbor information, fewer control packages and a novel holding time mechanism, CDTP reduces end-to-end latency, energy consumption and multi-copy problems simultaneously. The simulation results show that CDTP can perform better in a dense UWSN environment.

## Figures and Tables

**Table 1:** The competitor set of n.

A	B	C	D	E	F
$N_{1}$,$N_{2}$	$N_{2}$,$N_{5}$	$N_{6}$,$N_{7}$	$N_{3}$,$N_{4}$	$N_{5}$,$N_{6}$	$N_{6}$,$N_{7}$,$N_{8}$

## References

[1] Akyildiz I.F., Pompili D., Melodia T. (2005). Underwater acoustic sensor networks: Research challenges. Ad Hoc Netw..

[2] Bharghavan V., Demers A., Shenker S., Zhang L. (1994). MACAW: A media access protocol for wireless LAN’s. ACM SIGCOMM Comput. Commun. Rev..

[3] Jiang S. (2018). State-of-the-Art Medium Access Control (MAC) Protocols for Underwater Acoustic Networks: A Survey Based on a MAC Reference Model. IEEE Commun. Surv. Tutor..

[4] Gopi S., Kannan G., Chander D., Desai U.B., Merchant S. Pulrp: Path unaware layered routing protocol for underwater sensor networks. Proceedings of the 2008 IEEE International Conference on Communications.

[5] Shah G. A Survey on Medium Access Control in Underwater Acoustic Sensor Networks. Proceedings of the International Conference on Advanced Information Networking and Applications Workshops.

[6] Peng Z., Zhou Z., Cui J.H., Shi Z. Aqua-Net: An underwater sensor network architecture: Design, implementation, and initial testing. Proceedings of the OCEANS 2009.

[7] Yan H., Shi Z.J., Cui J.H. DBR: Depth-based routing for underwater sensor networks. Proceedings of the International Conference on Research in Networking.

[8] Liu J., Yu M., Wang X., Liu Y., Wei X., Cui J. (2018). RECRP: An underwater reliable energy-efficient cross-layer routing protocol. Sensors.

[9] Jornet J.M., Stojanovic M., Zorzi M. Focused beam routing protocol for underwater acoustic networks. Proceedings of the third ACM international workshop on Underwater Networks.

[10] Khan A., Altowaijri S.M., Ali I., Rahman A.U. (2019). Reliability-Aware Cooperative Routing with Adaptive Amplification for Underwater Acoustic Wireless Sensor Networks. Symmetry.

[11] Dou F., Peng Z. On-demand Pipelined MAC for Multi-hop Underwater Wireless Sensor Networks. Proceedings of the 10th International Conference on Underwater Networks & Systems.

[12] Bouabdallah F., Boutaba R. A Distributed OFDMA Medium Access Control for Underwater Acoustic Sensors Networks. Proceedings of the 2011 IEEE International Conference on Communications (ICC).

[13] Wahid A., Lee S., Jeong H.J., Kim D. Eedbr: Energy-efficient depth-based routing protocol for underwater wireless sensor networks. Proceedings of the International Conference on Advanced Computer Science and Information Technology.

[14] Guan Q., Ji F., Liu Y., Yu H., Chen W. (2019). Distance-vector-based opportunistic routing for underwater acoustic sensor networks. IEEE Internet Things J..

[15] Su Y., Fan R., Fu X., Jin Z. (2019). DQELR: An Adaptive Deep Q-Network-Based Energy- and Latency-Aware Routing Protocol Design for Underwater Acoustic Sensor Networks. IEEE Access.

[16] Ullah U., Khan A., Altowaijri S.M., Ali I., Rahman A.U., Vijay K.V., Ali M., Mahmood H. (2019). Cooperative and Delay Minimization Routing Schemes for Dense UnderwaterWireless Sensor Networks. Symmetry.

[17] Zhang J., Cai M., Han G., Qian Y., Shu L. (2020). Cellular Clustering-Based Interference-Aware Data Transmission Protocol for Underwater Acoustic Sensor Networks. IEEE Trans. Veh. Technol..

[18] Hsu C.C., Lai K.F., Chou C.F., Lin C.J. ST-MAC: Spatial-Temporal MAC Scheduling for Underwater Sensor Networks. Proceedings of the IEEE INFOCOM 2009, Rio de Janeiro.

[19] Zhang R., Cheng X., Cheng X., Yang L. (2017). Interference-Free Graph Based TDMA Protocol for Underwater Acoustic Sensor Networks. IEEE Trans. Veh. Technol..

[20] Gopi S., Kannan G., Desai U.B., Merchant S. Energy optimized path unaware layered routing protocol for underwater sensor networks. Proceedings of the IEEE GLOBECOM 2008—2008 IEEE Global Telecommunications Conference.

[21] Al-Bzoor M., Zhu Y., Liu J., Reda A., Cui J.H., Rajasekaran S. Adaptive power controlled routing for underwater sensor networks. Proceedings of the International Conference on Wireless Algorithms, Systems, and Applications.

[22] Gopi S., Govindan K., Chander D., Desai U.B., Merchant S. (2010). E-PULRP: Energy optimized path unaware layered routing protocol for underwater sensor networks. IEEE Trans. Wirel. Commun..

[23] Liu X., Liu P., Long T., Lv Z., Tang R. An efficient depth-based forwarding protocol for underwater wireless sensor networks. Proceedings of the 2018 IEEE 3rd International Conference on Cloud Computing and Big Data Analysis (ICCCBDA).

[24] Luo Y., Pu L., Peng Z., Cui J.H. Dynamic control channel MAC for underwater cognitive acoustic networks. Proceedings of the IEEE Infocom—The IEEE International Conference on Computer Communications.

[25] Bouabdallah F., Zidi C., Boutaba R., Mehaoua A. (2019). Collision Avoidance Energy Efficient Multi-Channel MAC Protocol for UnderWater Acoustic Sensor Networks. IEEE Trans. Mob. Comput..

[26] Akyildiz I.F., Pompili D., Melodia T. (2004). Challenges for efficient communication in underwater acoustic sensor networks. ACM Sigbed Rev..

[27] Cui J.H., Kong J., Gerla M., Zhou S. (2005). Challenges: Building scalable and distributed Underwater Wireless Sensor Networks (UWSNs) for aquatic applications. Channels.

[28] Liu J., Wang Z., Peng Z., Cui J., Fiondella L. Suave: Swarm underwater autonomous vehicle localization. Proceedings of the IEEE INFOCOM 2014—IEEE Conference on Computer Communications.

[29] Lee U., Kong J., Gerla M., Park J.S., Magistretti E. (2007). Time-critical underwater sensor diffusion with no proactive exchanges and negligible reactive floods. Ad Hoc Netw..

[30] Brekhovskikh L.M., Lysanov Y.P., Beyer R.T. (1991). Fundamentals of Ocean Acoustics.

[31] Brekhovskikh L., Lysanov Y.P. (2004). Fundamentals of Ocean Acoustics.

[32] Mason S.F., Berger C.R., Zhou S., Willett P. (2008). Detection, synchronization, and Doppler scale estimation with multicarrier waveforms in underwater acoustic communication. IEEE J. Sel. Areas Commun..

[33] Xie P., Zhou Z., Peng Z., Yan H., Hu T., Cui J.H., Shi Z., Fei Y., Zhou S. Aqua-Sim: An NS-2 based simulator for underwater sensor networks. Proceedings of the OCEANS 2009.

[34] Ma Z., Guan Q., Ji F., Yu H., Chen F. An efficient and low-signaling opportunistic routing for underwater acoustic sensor networks. Proceedings of the International Conference on Information Science and Applications.

[35] Xie P., Cui J. R-MAC: An Energy-Efficient MAC Protocol for Underwater Sensor Networks. Proceedings of the International Conference on Wireless Algorithms, Systems and Applications (WASA 2007).

